# Development of Gut Microbiota in the First 1000 Days after Birth and Potential Interventions

**DOI:** 10.3390/nu15163647

**Published:** 2023-08-20

**Authors:** Alexandru Cosmin Pantazi, Adriana Luminita Balasa, Cristina Maria Mihai, Tatiana Chisnoiu, Vasile Valeriu Lupu, Mustafa Ali Kassim Kassim, Larisia Mihai, Corina Elena Frecus, Sergiu Ioachim Chirila, Ancuta Lupu, Antonio Andrusca, Constantin Ionescu, Viviana Cuzic, Simona Claudia Cambrea

**Affiliations:** 1 Pediatrics Department, Faculty of Medicine, “Ovidius” University, 900470 Constanta, Romaniaadriana_balasa@yahoo.com (A.L.B.);; 2 Pediatrics Department, County Clinical Emergency Hospital of Constanta, 900591 Constanta, Romania; 3 Pediatrics Department, “Grigore T. Popa” University of Medicine and Pharmacy, 700115 Iasi, Romania; 4 Faculty of Medicine, “Ovidius” University, 900470 Constanta, Romaniasergiu.chirila@univ-ovidius.ro (S.I.C.);; 5 Infectious Diseases Department, Faculty of Medicine, “Ovidius” University, 900470 Constanta, Romania

**Keywords:** microbiota, colonization, children, breastfeeding

## Abstract

The first 1000 days after birth represent a critical window for gut microbiome development, which is essential for immune system maturation and overall health. The gut microbiome undergoes major changes during this period due to shifts in diet and environment. Disruptions to the microbiota early in life can have lasting health effects, including increased risks of inflammatory disorders, autoimmune diseases, neurological disorders, and obesity. Maternal and environmental factors during pregnancy and infancy shape the infant gut microbiota. In this article, we will review how maintaining a healthy gut microbiome in pregnancy and infancy is important for long-term infant health. Furthermore, we briefly include fungal colonization and its effects on the host immune function, which are discussed as part of gut microbiome ecosystem. Additionally, we will describe how potential approaches such as hydrogels enriched with prebiotics and probiotics, gut microbiota transplantation (GMT) during pregnancy, age-specific microbial ecosystem therapeutics, and CRISPR therapies targeting the gut microbiota hold potential for advancing research and development. Nevertheless, thorough evaluation of their safety, effectiveness, and lasting impacts is crucial prior to their application in clinical approach. The article emphasizes the need for continued research to optimize gut microbiota and immune system development through targeted early-life interventions.

## 1. Introduction

The first 1000 days of a child represent a critical window for the maturation of the immune system and the establishment of gut microbiota [1]. This simultaneous development has caught the attention of immunology researchers, making it an area of study that is both intriguing and captivating. The human being lives in harmony with microbiota, which is made up of not only bacteria but also viruses and fungi [2]. These microorganisms are present throughout the human body in different sites such as the skin, mouth, nasopharynx, and intestine [2,3]. Identifying the bacterial composition of the prenatal meconium has been challenging due to the potential of microbiological contamination [4,5,6]. It is widely documented that the process of microbial colonization starts quickly after birth, as evidenced by numerous studies [7,8]. During the initial 1000 days after birth, breastfeeding influences the intestinal microbiome, which is dominated by *Bifidobacterium* [1]. This group of bifidobacteria includes *Bifidobacterium bifidum*, *Bifidobacterium breve*, and *Bifidobacterium longum* spp. [9,10]. The microbiome undergoes significant transformations during two crucial developmental phases early in life: from birth until weaning, and then during the transition from weaning into early adulthood. These modifications are driven by the diversification of the diet, resulting in considerable changes to the microbial composition [11]. The variance in microbiome configurations depends on the infant’s genetics plus the infant’s environmental properties, including humidity, pH, nutrients, and oxygenation [2]. This article aims to provide insights into the changes that occur in the infant’s gut microbiota during the first 1000 days after birth and potential therapies.

## 2. Development of Maternal Intestinal Microbiota during Pregnancy

During pregnancy, the mother’s body experiences numerous physiological and metabolic changes that intend to provide an optimal intrauterine environment for the developing fetus and to ensure proper growth, development, and accommodation [12]. These gestational changes include endocrine, immunological, and metabolic modifications that promote a pro-inflammatory state, leading to specific changes in the maternal microbiota across different body sites, such as the vaginal area, intestine, and oral cavity [13]. The development and growth of the fetus are significantly influenced by the in utero environment and fetal-maternal interactions [14]. According to a study, around 20% of stunting cases are due to preterm birth, small-for-gestational age (SGA), or both, which have their origins in the uterus [15]. Multiple studies indicate that the transmission of bacteria from the mother influences the development and expansion of a healthy neonatal microbiome, which can impact infant growth [16,17], immune system maturation [18,19,20], and neurodevelopment [21,22]. Therefore, it is vital to maintain a healthy intestinal microbial composition during pregnancy. It was found that the microbiota composition of pregnant women is different in many aspects from non-pregnant women and changes throughout pregnancy [23,24]. The gut microbiota in late pregnancy is reduced compared with the first trimester, with a decrease in the number of *Firmicutes* and an increase in the presence of *Proteobacteria*, *Actinobacteria*, and *Streptococcus* [23]. These shifts in the gut microbiota produce in pregnant women susceptibility to gestational diabetes and an increase the incidence of macrosomia [23]. Additionally, another study reported that the reduced alpha-diversity of the gut microbiota in late pregnancy is linked to adverse outcomes such as low birth weight [25]. In the early phase after birth, the infant microbiota begins to resemble that of an adult with a predominance of *Firmicutes*, *Bacteroides* [26], *Bifidobacterium*, *Parabacteroides*, *Escherichia*, *Shigella*, *Lactobacillus*, and *Prevotella* [27]. During this period, the microbiota undergoes further diversification and becomes more stable. This is an important phase, as it sets the stage for long-term health outcomes [28,29].

## 3. First Gut Microbiota of Infants

Delivery plays a crucial role in establishing the initial colonization of the infant’s gut microbiota. The skin, mouth, and intestine of newborns who were delivered vaginally contain *Lactobacillus*, which is one of the most abundant microorganisms of the maternal vaginal flora [30]. After birth, the first microorganisms of the infant’s body come from the mother’s microbiota found in the vaginal area, feces, breast milk, mouth, and skin, as well as the surrounding environment [31,32,33]. During the initial years of life, the microbial population that inhabits the gastrointestinal tract includes facultative anaerobic bacteria. However, in the weeks to come, these early colonizers are gradually displaced by conventional anaerobic bacteria that eventually come to dominate the intestinal microbiota [34]. Immediately after birth, the newborn’s intestinal microbiota is firstly overpopulated by the presence of *Enterobacteriaceae* and *Staphylococcus* [35], but later it is replaced by *Bifidobacterium* and some lactic acid bacteria [36]. This *Bifidobacterium*-dominated microbiota, also known as “*Bifidus flora*”, remains until the introduction of complementary food [37,38]. As the infant approaches weaning, the relative abundance of *Bacteroides* gradually increases, leading to the competitive exclusion of *Bifidobacterium* within the intestinal microbiota. After weaning, the *Bifidus flora* is replaced by adult-type microorganisms, which mainly include bacteria such as *Bacteroides*, *Prevotella*, *Ruminococcus*, *Clostridium*, and *Veillonella* [37]. By three years of age, the gut microbiota becomes similar to that of an adult [39]. The gut microbiota’s functions also change significantly before and after the introduction of solid foods. In the first year of life, the early microbiota is enriched with bacteria that can utilize lactate. Solid food promotes the growth of bacteria that can use a wider variety of carbohydrates, synthesize vitamins, and degrade xenobiotics [37,39,40]. By this period, the infant’s gut has been exposed to a variety of environmental factors such as breastfeeding, type of delivery and exposure to antibiotics, which can have a significant impact on the further development of the infant’s intestinal flora [41,42,43].

## 4. Factors Influencing the Intestinal Microbiota during the First 1000 Days after Birth

### 4.1. Maternal Factors

In infants with normal gestational age, microbiota develops more quickly towards that of an adult when compared to preterm infants [44]. Premature infants are characterized by lower diversity of the intestinal microbiota compared to full-term infants [45]. The type of birth, especially vaginal delivery, determines in newborns microbial species from the vaginal area of the mother and perianal regions, including *Lactobacillus*, *Prevotella*, or *Sneathia* spp. [46], while infants who are delivered through caesarian section have limited exposure to these bacteria [47]. The maternal vaginal microbiota is an important contributing factor. When women are pregnant, microbial variety within the vaginal microbiota decreases; on the other hand, the abundance of *Lactobacillus* spp. increases, potentially strengthening their defensive function [48,49]. Besides vaginal bacteria, uterine and placental bacteria may confer potential benefits to both the developing fetus and the newborn. Specifically, these microbes may foster tolerance towards microorganisms that promote postnatal well-being, including those affiliated with the *Lactobacillus genus* [50].

However, some studies suggest that dysbiosis that occurs at the time of pregnancy leads to an increased risk of premature birth [51]. A relation between microbiota and preterm births was observed [52]. The study found that women who delivered prematurely exhibited significantly different vaginal microbiota compared with women who gave birth to full-term infants. Explicitly, women who gave birth preterm had decreased levels of *Lactobacillus*. These findings suggest that the composition of the vaginal microbiota may be a useful indicator of the risk of preterm birth. Another possible factor is maternal health status; this includes obesity, gestational diabetes, and inflammation, which can participate in the instability of the constituents and composition of the infant’s intestinal microbiota, as observed by the analysis of the newborn meconium. Additionally, maternal inflammation during pregnancy has been associated with an increased risk of gut dysbiosis in the infants [53,54,55]. It was observed that maternal diet could influence the microbiota, as a high-fat diet in mothers affects the initial colonization of bacteria [56]. When the mother consumed a high-fat diet, the meconium microbiome of the neonate was significantly depleted in *Bacteroides*, and this depletion continued until the infant was 6 weeks old [56].

The neonatal gut microbiota is significantly influenced by a maternal high-fat diet and not just by maternal obesity [57,58,59]. Microbiota in maternal milk may come from the mother’s gut microbiota [60,61]. Therefore, if the mother’s gut microbiota is imbalanced due to an unhealthy diet, this could potentially be transferred to the milk and affect the infant’s gut microbiome during breastfeeding [58]. However, there is currently limited research on the long-term effects of maternal lifestyle and health during pregnancy and breastfeeding on the infant’s gut microbiota.

Exposure to antibiotics during pregnancy has a significant impact on the microbiome, leading to a reduction in microbial load and changes in composition, as well as long-term effects on the development of the infant gut microbiome [62]. Another study has shown that antibiotics can have an impact on the microbiome of breast milk, with high levels of *Bifidobacterium* found in breast milk from mothers who did not receive antibiotics intrapartum [63]. Furthermore, antibiotic use during lactation can result in a decrease in the microbial community of breast milk, including *lactobacilli* and *bifidobacteria*, and is associated with lower bacterial diversity in breast milk [64,65].

### 4.2. Environmental Factors

It is widely recognized that the mode of delivery plays a crucial role in determining the initial bacterial colonization of infants [46]. Children born via caesarian section have a risk for the development of specific diseases such as obesity, allergy, asthma, and atopy possibly due to altered immune development [66,67]. Infants born vaginally are colonized by bacteria that are normally located in the maternal vagina [46]. On the other hand, infants born via caesarian section are colonized by bacteria that are similar to those present on the maternal skin and in the oral cavity [47,49]. Furthermore, extensive studies that have monitored the composition of microbiota in infants from birth until 2 years of age suggested a correlation between delivery by caesarian section and delayed colonization of the *Bacteroidetes phylum*, as well as lower overall microbial diversity up to 2 years of age [68]. Moreover, at 7 years of age, differences were observed between the microbiotas of infants born via caesarian section and those born vaginally [69]. The microbial distinctions observed in children delivered via caesarian section encompass heightened colonization rates of *C. difficile* and other *clostridia*, alongside diminished levels of *lactobacilli*, *bifidobacteria*, *bacteroides*, and *E. coli* [69]. The bacterial microbiota is influenced by gestational age [70]. Premature neonates are often subjected to heightened antibiotic exposure and frequently experience prolonged hospital stays, mechanical ventilation, and parenteral nutrition. Such conditions have the potential to induce enduring modifications to the typical colonization and developmental mechanisms of the gut microbiota [2,71]. Adopting a Mediterranean-style diet, supplementing with probiotics and prebiotics, and breastfeeding are all crucial for maintaining a diverse gut microbiota. These practices also provide newborns with essential nutrients and can help reduce the risk of developing allergies [72].

Breastfeeding can be considered among the most crucial factors in the normal development of infant microbiota. The type of nutrition, whether through breastfeeding or formula, significantly affects the composition of gut microbiota in the first days after birth. Human milk is rich in nutrients such as fats, proteins, and carbohydrates, and contains immunoglobulins and endocannabinoids. Constituents of breast milk are responsible for selecting the types of bacteria that colonize the gastrointestinal tract. For instance, immune regulatory cytokines like TGF-β and IL-10 in breast milk facilitate the host immune system’s tolerance to intestinal bacteria and enhance the production of IL-10 in infants [73,74]. The oligosaccharides in human milk (HMO), particularly galactooligosaccharide (GOS), are incompletely broken down in the small intestine and instead primarily fermented in the colon by *Bifidobacterium*, generating short-chain fatty acids [75,76]. Research by Matsuki et al. [35] has shown that the growth of *Bifidobacterium* in infants’ guts leads to a decrease in the number of HMOs present in feces, with a concurrent increase in acetic acid and lactic acid. These results imply that HMOs have a beneficial impact on the development of a *Bifidobacterium*-dominated gut microbiota [35]. It is recommended to encourage mothers to breastfeed their infants, according to a cross-sectional study involving 1008 mothers with children aged 9 to 14 months, in which factors influencing the duration of breastfeeding were investigated [77]. The study findings revealed that perinatal education and mother-baby-friendly hospitals were associated with extended breastfeeding duration. Notably, the conventional teaching of pregnant mothers and proactive engagement in perinatal education emerged as crucial factors in promoting the duration of breastfeeding. The formula-fed infants show an abundance of *Bacteroides*, *Clostridium coccoides*, and *Lactobacillus* [78]. Other studies found that the fecal analysis from formula-fed infants is most likely to contain *staphylococci*, *Escherichia coli*, and *clostridia* [79,80]. However, in recent times, milk formula has undergone enhancements, including the addition of certain oligosaccharides, to enable the development of a microbiota rich in *Bifidobacterium* in infants [81].

Administering antibiotics during the early stages after birth can have a significant impact on the development of gut microbiota. It causes a shift in the composition of gut microbiota towards a higher proportion of *Proteobacteria* and a lower proportion of *Actinobacteria* populations [82,83]. This also results in reduced overall diversity of the infant’s microbiota and a selection of drug-resistant bacteria [84,85]. In a study by Tanaka et al. [82], the impact of early postnatal exposure to antibiotics on the development of intestinal bacteria was observed in 26 infants. Among these infants, five were given antibiotics orally for the first four days after birth, while three received antibiotics intravenously before delivery. The study discovered that the infants who were exposed to antibiotics had fewer types of bacteria, with reduced colonization of *Bifidobacterium* and abnormal colonization of *Enterococcus* during the first week. Additionally, an overgrowth of *enterococci* and a lack of growth of *Bifidobacterium* were observed in the antibiotic-exposed group. One month later, the antibiotic-exposed group had a higher population of *Enterobacteriaceae* compared with the control group. These findings suggest that exposure to antibiotics at an early stage of life can significantly impact the development of neonatal intestinal microbiota [82]. Solid foods represent another influencing factor for the composition of gut microbiota in infants. When solid foods containing indigestible carbohydrates are introduced into an infant’s diet, their gut quickly acquires a functional gene pool that is largely dominated by carbohydrate metabolism genes, similar to that of an adult [39]. Factors affecting the infant’s gut microbiota during the first 1000 days after birth are described in Table 1.

**Table 1:** Summary of the factors affecting the infant’s intestinal microbiota during the first 1000 days after birth.

Category	Factor	Description
Maternal	Gestational Age	Full-term infants develop microbiota more quickly than preterm infants, who have lower intestinal microbiota diversity [44,45].
Maternal	Mode of Delivery	Vaginal delivery exposes newborns to maternal vaginal and perianal microbes, while caesarian section limits this exposure [46,47].
Maternal	Maternal Vaginal Microbiota	Microbial variety in the vaginal microbiota decreases during pregnancy, potentially impacting the infant’s microbiome [48,49]. Dysbiosis may increase the risk of premature birth [52].
Maternal	Maternal Health Status	Obesity, gestational diabetes, and inflammation can impact the infant’s intestinal microbiota and increase the risk of gut dysbiosis [53,54,55].
Maternal	Maternal Diet	A high-fat diet can impact the initial colonization of bacteria in offspring, regardless of the mother’s obesity status [56,57,58].
Maternal	Antibiotic Exposure During Pregnancy	Antibiotics during pregnancy can reduce microbial load and alter the composition of the infant’s gut microbiome. They can also impact the breast milk microbiome [63,64,65].
Environmental	Gestational Age	Premature infants exposed to high levels of antibiotics and long hospital stays have altered gut microbiota development [2,70,71].
Environmental	Feeding Method	Breastfeeding significantly impacts the formation of the gut microbiota, promoting the growth of *Bifidobacterium* [73,74]. Formula feeding results in a different gut microbiota composition, but recent improvements aim to encourage *Bifidobacterium* growth [78,79,80].
Environmental	Antibiotic Exposure	Early exposure to antibiotics leads to altered gut microbiota composition, reduced overall diversity, and a selection for drug-resistant bacteria [81,82,83,84,85].
Environmental	Introduction of Solid Foods	The introduction of solid foods containing indigestible carbohydrates affects the infant’s gut microbiota, leading to a functional gene pool similar to that of an adult [39,40,47].

## 5. Intestinal Microbiota in Health and Disease

In recent years, the role of intestinal microecosystems has been widely studied in health and disease, with numerous studies highlighting the significant impact that the gut microbiota can have on overall health. Recent studies have shown that the intestinal microbiota plays a crucial role in immune system development [7,18,86,87,88]. By interacting with the immune cells of the gut, the microbiota helps to establish immune tolerance and prevent the development of inflammatory and autoimmune disorders [89]. The bacterial species *Bacteroides fragilis* has been shown to promote the development of regulatory T cells (Treg), which are essential for maintaining immune tolerance [90]. Short-chain fatty acids (SCFAs) produced by gut bacteria play a crucial role in linking the gut microbiota and immune system maturation. A recent study examined fecal samples from 100 mothers and their children to investigate changes in gut microbiota and SCFA composition as children age [91]. The results revealed positive correlations between certain bacteria and specific SCFAs, suggesting that these changes may have implications for preventing early-life immunological disorders. A novel area of interest in this field is the impact of maternal microbiota on the offspring’s immune system development. Recent studies have suggested that vertical transmission of maternal microbiota, particularly during vaginal birth, could be essential for the proper development of the neonatal immune system [92]. However, further research is needed to elucidate the specific mechanisms by which the maternal microbiota influences the immune system and to identify potential therapeutic targets for immune-related disorders. Furthermore, a possible relationship between heart failure and the microbiota was suggested [93] due to the association of progression of heart failure with decreased bacterial diversity, a higher abundance of potentially detrimental bacteria, a reduced production of short-chain fatty acids by gut bacteria, and increased intestinal permeability allowing microbial translocation and the passage of bacterial-derived metabolites into the bloodstream.

Necrotizing enterocolitis (NEC) is a condition marked by inflammation and necrosis of the intestine, which can advance to systemic infection, multiorgan failure, and eventual mortality [94]. Numerous microorganisms have been identified as potential contributors to the pathogenesis of NEC [95]. Additionally, fungi such as *Candida* spp. may also play a role in the pathogenesis of necrotizing enterocolitis [96]. The intestinal microbial composition of infants who develop NEC differs significantly from those who do not, which has been reported repeatedly in the literature [97,98]. Another study has shown that alterations in fecal bacteria can be detected as early as 72 h before the onset of NEC [99]. Allergic diseases have been associated with dysbiosis in children [100,101,102,103,104]. Recent studies have emphasized the significant role of the gut microbiota in influencing the pathogenesis of respiratory pathology, by the “gut-lung axis” phenomenon. Dysbiosis of the respiratory microbiota can disrupt immune regulation, increasing the risk of respiratory infections and allergic conditions. Moreover, alterations in microbial composition can influence immune system maturation and the delicate balance between pro-inflammatory and anti-inflammatory responses in the respiratory tract [105,106,107], while other studies have explained the connection between intestinal microbiota and allergies [108]. A study showed that fungi play a role in gut microbiome ecology and host immune function [109]. Fungal colonization causes significant changes in the bacterial community and has a separate effect on immune development. Although fungi alone may not cause colitis, co-colonization with bacteria and fungi increased inflammation in the colon. The findings suggest that therapies aimed at modulating early-life microbiomes should consider the role of fungi.

Emerging evidence suggests that the gut microbiota may also have a significant impact on the development of neurological disorders during early life [110]. The gut-brain axis is a unique communication pathway that connects the central nervous system with the gastrointestinal tract, and the gut microbiota plays a key role in modulating this communication [111].

Altered gut microbiota composition has been observed in children with autism spectrum disorder (ASD), and some researchers have proposed that dysbiosis could contribute to the behavioral symptoms of ASD [112]. A novel area of interest is the potential use of microbiota-targeted interventions, such as fecal microbiota transplantation, in the treatment of ASD and other neurological disorders [113]. Nonetheless, further research is needed to establish the safety and efficacy of such interventions.

The intestinal microbiota has also been implicated in the development of obesity during early life [12,42,114]. An imbalance of the intestinal microecosystem characterized by a diminished abundance of *Bacteroides* and an increased abundance of *Firmicutes* is correlated with increased adiposity and weight gain [115]. Considering the pivotal function of the microbiota in immunological responses, it can be postulated that the phenomenon of dysbiosis might engender conditions more propitious for the proliferation and dispersion of deleterious microorganisms, such as *Shigella* spp. This hypothesis garners support from a retrospective investigation spanning a ten-year period that encompassed 376 *Shigella*-afflicted patients [116]. The research discerned a heightened susceptibility to *Shigella* spp. in children below the age of five years. Further, it underscored the substantial role played by environmental factors such as ambient temperature, humidity, and precipitation in modulating the prevalence of *Shigella* spp. infections. These environmental factors may trigger dysbiosis, leading to increased vulnerability of the child’s intestine to *Shigella species.* A recent review showed that while the exact mechanisms are still not entirely clear on how post-infectious irritable bowel syndrome (PI-IBS) starts, alterations in gut microbiota, immune responses, and gut-brain interactions likely contribute to the development of PI-IBS [117], suggesting the connection between microbiota and PI-IBS.

## 6. Conventional Treatment for Mitigating Dysbiosis in Infants

Consuming live microorganisms, referred to as probiotics, in appropriate quantities has been shown to offer health benefits to the host [118]. These products have demonstrated efficacy in preventing and addressing a variety of gut microbiota-related disorders, such as antibiotic-associated diarrhea [119], inflammatory bowel disease [120], and allergies [121,122]. A meta-analysis which reviewed ten studies on allergic rhinitis [122] found that five of them reported a substantial reduction in symptom scores and an enhancement in the quality of life for patients with allergic rhinitis, suggesting a positive impact of probiotics. *Bifidobacterium* and *Lactobacillus*, essential microorganisms during early childhood, appeared effective in treating allergic rhinitis in the study. Further research is needed to substantiate these findings. Prebiotics, non-digestible components found in food like fibers and carbohydrates, selectively encourage the growth and activity of the human gut’s micro-ecosystem [123]. These components have been investigated as a possible treatment for gut-microbiota-related pathologies. Prebiotics have also demonstrated a positive effect in reducing the risk of allergies; for example, a study on infants showed that prebiotic supplementation lowered the risk of atopic dermatitis by 50% compared with placebo treatment [124].

Fecal microbiota transplantation is a technique that can normalize the gut microbiota of affected individuals by replacing it with a healthy individual’s microecosystem [125]. This method has proven to be effective in the treatment of various conditions, such as recurrent *Clostridium difficile* infection [126], inflammatory bowel disease [127], and even obesity [128]. Breastfeeding has been identified as a factor that significantly impacts the development of the microbiota during an infant’s early life, as it provides essential nutrients [129,130].

Finally, associations between microbiota composition and digestive disorders such as esophageal cancer, gastritis, celiac disease, Helicobacter pylori infection, and systemic lupus erythematosus was found [131,132,133,134,135,136,137].

## 7. Exploring Promising Interventions for Mitigating Dysbiosis in Early Childhood

### 7.1. Prebiotic and Probiotic-Enriched Hydrogels

Hydrogels are three-dimensional networks of hydrophilic polymers capable of retaining large amounts of water [138]. They have been explored as drug-delivery systems for various applications [139]. One of these applications is the use of these hydrogels as a carrier for probiotics, which has been found in the literature on many occasions [140,141]. A promising intervention would involve the development of hydrogels containing prebiotics and probiotics to promote the growth of beneficial gut bacteria and counteract negative influences on the microbiota. The viability of these beneficial bacteria can be compromised by various factors, including the acidic environment of the gastric juice, which has a severe impact on bacteria [142,143]. By encapsulating probiotic cells within a hydrogel, a physical barrier can be created to protect them from harmful environmental conditions, thereby enhancing their survival rate. Notably, hydrogels have been found to improve the viability of probiotic bacteria not only under gastrointestinal conditions but also during storage at various temperatures or exposure to heat treatment [144]. Thus, the utilization of hydrogels appears to be a viable and optimistic strategy to amplify the effectiveness of probiotics, thereby securing their advantageous impacts on the health of infants. However, before the use of prebiotic- and probiotic-enriched hydrogels can become a viable intervention, several important factors must be considered, such as the biocompatibility and safety of these hydrogels.

### 7.2. Gut Microbiota Transplantation (GMT) during Pregnancy

Another promising novel intervention for optimizing the maternal gut microbiota during pregnancy is gut microbiota transplantation (GMT). While fecal microbiota transplantation (FMT) is a well-established treatment for recurrent *Clostridioides difficile* infection (CDI) in adults [145,146,147], GMT during pregnancy could represent a promising approach to enhance pregnancy outcomes and minimize the risk of adverse effects on the infant’s microbiota. A case study conducted by Saeedi et al. [148] elaborated a promising effect of FMT for the treatment of *Clostridioides difficile*. However, guidelines published by the International Consensus Conference on fecal microbiota transplantation stated that FMT should be avoided unless it was strictly needed for pregnant women with severe CDI infection in whom standard therapy has been found ineffective [125]. The lack of data regarding the safety and efficacy of GMT during pregnancy raises concerns about the risks associated with this intervention. The safety of GMT during pregnancy must be thoroughly evaluated to ensure that its risks do not exceed its health benefits for the mother or developing fetus. This should include the potential long-term effects of GMT on the infant’s microbiota, and overall health must be carefully studied.

### 7.3. Microbial Ecosystem Therapeutics (MET) for Infants

Microbial ecosystem transplantation (MET) is an advanced and more precise approach than fecal microbiota transplantation (FMT). Which involves the transplantation of processed fecal matter from a healthy donor, MET involves the purification and cultivation of selected beneficial bacteria from the sample, which results in a well-defined and stable microbial ecosystem that can be transplanted into the recipient [149,150]. An innovative application of MET could be the development of age-specific formulations for infants during the first 1000 days after birth. These formulations could be tailored to meet the specific needs of the infant’s gut microbiota, promoting healthy development, and preventing or treating dysbiosis. By supporting immune and metabolic development, these age-specific MET formulations could have long-term benefits for infant health. Further investigation is needed to assess the safety, efficacy, and long-term consequences of MET in infants. However, the potential benefits of this approach make it a promising avenue for future research in the field of infant gut microbiota therapeutics.

### 7.4. Gut-Microbiota-Targeted CRISPR Therapies

Recent advances in gene-editing technologies have paved the way for novel interventions that specifically target harmful bacteria in the gut microbiota without affecting beneficial ones. The CRISPR/Cas9 system is a promising candidate for developing gut-microbiota-targeted therapies [151]. The idea behind this approach is to engineer CRISPR systems to target and eliminate specific bacteria, thus promoting a healthy gut microbiota in the first 1000 days after birth. This approach could potentially reduce the risk of various health issues associated with dysbiosis, such as metabolic disorders and immune dysfunction. Furthermore, research is ongoing on editing the microbiota using CRISPR [152,153]. However, several challenges need to be overcome before CRISPR-based therapies can be translated into clinical practice, including the efficient delivery of the CRISPR systems to the gut, specificity of targeting, and off-target effects. Further research is needed to determine the feasibility and safety of this approach in humans. Some interventions for mitigating dysbiosis in the first 1000 days after birth are described in Table 2.

## 8. Conclusions

In conclusion, the first 1000 days after birth are crucial for the development of the infant’s gut microbiota and immune system. The microbiome undergoes significant transformations during this period, driven by changes in diet and environmental factors. Disturbances to the microbiota during this window can have long-term effects on the infant’s health, including the development of inflammatory and autoimmune disorders, neurological disorders, and obesity. Maternal factors, including gestational age, mode of delivery, maternal vaginal microbiota, maternal health status, maternal diet, and exposure to antibiotics during pregnancy and lactation, influence the infant’s gut microbiota. Environmental factors, such as mode of delivery, gestational age, and breastfeeding, also play an important role. Research continues to explore early-life interventions to optimize gut microbiota and immune system development. Maintaining a healthy intestinal microbial community during pregnancy and infancy is vital for the long-term health outcomes of the infant. Potential novel approaches, such as prebiotic- and probiotic-enriched hydrogels, GMT during pregnancy, age-specific MET formulations, and gut microbiota-targeted CRISPR therapies, offer promising avenues for future research and development. However, rigorous investigation of their safety, efficacy, and long-term consequences is necessary before their implementation in clinical practice. Further research is required to fully comprehend the complicated relationship between the intestinal microecosystem and the developing infant and to develop effective interventions to promote optimal gut health during the infant years.

## Figures and Tables

**Table 2:** Summary of the promising interventions for mitigating dysbiosis in the first 1000 after birth.

Intervention	Description	Potential Benefits	Challenges/Considerations
Prebiotic and probiotic-enriched hydrogels	Development of hydrogels containing prebiotics and probiotics to promote the growth of beneficial gut bacteria and counteract negative influences on the microbiota	Enhanced survival rate of probiotics, improved effectiveness of probiotics, promotion of healthy intestinal microbiota	Thorough evaluation of biocompatibility and safety of hydrogels, rigorous testing in clinical trials
Gut microbiota transplantation (GMT) during pregnancy	Transfer of select beneficial bacteria to optimize maternal gut microbiota during pregnancy and minimize the risk of adverse effects on the infant’s microbiota	Potential enhancement of pregnancy outcomes, potential minimization of the risk of adverse effects on infant’s microbiota	Lack of data on safety and efficacy, careful study of potential long-term effects on infant’s microbiota and overall health
Microbial ecosystem therapeutics (MET) for infants	Tailored formulations for infants during the first 1000 days after birth to meet specific needs of infant gut microbiota, promoting healthy development and preventing or treating dysbiosis	Potential support of immune and metabolic development, potential long-term benefits for infant health	A thorough investigation of safety, efficacy, and long-term consequences
Gut microbiota targeted CRISPR therapies	CRISPR/Cas9 system engineered to target and eliminate specific harmful bacteria, promoting healthy gut microbiota	Reduced risk of health issues associated with dysbiosis, such as metabolic disorders and immune dysfunction	The challenges of efficient delivery of CRISPR systems to gut microbiota, specificity of targeting, and off-target effects need to be overcome

## Data Availability

Data sharing not applicable.
